# Academic Staff’s Opinion on Their Job Satisfaction

**Published:** 2019-04

**Authors:** Rositsa DIMOVA, Rumyana STOYANOVA, Stanislava HARIZANOVA, Miglena TARNOVSKA, Donka KESKINOVA

**Affiliations:** 1. Department of Health Management and Health Economics, Faculty of Public Health, Medical University Plovdiv, Plovdiv, Bulgaria; 2. Department of Hygiene and Ecology, Faculty of Public Health, Medical University Plovdiv, Plovdiv, Bulgaria; 3. Department of Healthcare Management, Faculty of Public Health, Medical University Plovdiv, Plovdiv, Bulgaria; 4. Department of Applied and Institutional Sociology, University of Plovdiv Paisii Hilendarski, Plovdiv, Bulgaria

## Dear Editor-in-Chief

“Over the years, job satisfaction has received considerable attention from researchers. Investigations have predominantly been performed among business and hospital employees”. Few types of research have been conducted among medical academic staff and the impact of their specific work on job satisfaction (1).

The purpose of the study was to investigate the academic staff opinion on their working environment and job satisfaction. The cross-sectional survey was conducted among 370 academic staff members from six departments at the Medical University of Plovdiv in 2016.

This study was carried out with the co-operation of the Committee on Working Conditions and an Occupational Health and Safety expert. It was approved by the Vice Rector for Quality and Accreditation of the University. After giving their informed consent, the employees completed the questionnaire at work and returned it back to the authors.

The authors' survey tool was a 22-item standardized questionnaire. The independent variable was the working environment included factors such as working hours, job safety, job security, team-work, superior-subordinate communication, work organization, management styles and ways of handling conflicts within a workplace. The dependent variable was employee job satisfaction with a Likert-type response format of five points ranging from “1=complete disagreement” to “5=complete agreement”. In order to determine the impact of the working characteristics on the overall respondent satisfaction, the items related to the satisfaction of the received remuneration were excluded. In current study, we relied on the arguments of Leslie and McInnes, that the internal motivators play a greater role in academic staff job satisfaction than their remuneration (2, 3). Chandrasekar also argued that interpersonal relationships play a more dominant role in overall job satisfaction compared to wages (4). Data in our study was processed with the help of the statistical product IBM SPSS Statistics (ver. 22, Chicago, IL, USA).

The study questionnaire was tested for reliability and construct validity. Cronbach’s alpha for employee satisfaction was adequately high (0.749). Therefore, the accumulated data using the questionnaire has provided valuable information about the tested variable based on the opinions of employees. The present study found out that the investigated tools account for 57.7% of the total job satisfaction of the employees. The EFA revealed four factors related to employee satisfaction, including working activity organization (working hours and rest, work intensity); job safety; superior-subordinate communication and teamwork. The respondent’s answers showed that a considerable number of them 263 (71.7%) work in a risky work environment. Out of 15 listed risk factors, the respondents have indicated in the first place: mental strain −146 (39.5%) followed by ‘work with chemical agents and dust’ −140 (37.8%), and ‘work with biological hazards −133 (35.9%). Staff working at the Pharmacy and Dental Faculties are most frequently exposed to chemical factors (χ^2^ =61.389 *P*=0.001); regarding exposure to biological hazards- employees from the Faculties of Dental Medicine and Medicine are at a greater risk (χ^2^=83.916, *P* =0.001). Our study confirmed that ensuring health and safety working conditions is one of the most significant conditions for employee satisfaction (*P* =0.000). The results ascertained relatively high respondent evaluations regarding satisfaction with working activity organization, including work and rest balance, working day duration, work intensity, communication, and teamwork. The most common answer of the respondents associate to satisfaction with working activity organization was “agreement” (Table 1). A relation between satisfaction with assigned work intensity and satisfaction with staff numbers at the departments was ascertained (r_s_=0.529, *P* =0.001). Mutual respect, trust, as well as management support are essential for teamwork, shared sense of community, and empathy. Academic staff members receive greatest satisfaction out of their relationship with their immediate supervisor. Friendly relationships between the manager and other staff members are important reflections of job performance, regardless of the need to perform under pressure and overloaded work schedule (1, 5). The relevance to company culture, elements involving conflict handling and predominant communication styles were revealed in our study. Based on respondents’ opinion, the most commonly applied management style is democratic leadership. Similar to another study, our results revealed that democratic management style and good effective supervision results in higher employee satisfaction level (6). The nature of the cross-sectional design of our research is conductive to certain limitations. The study depicted the situation only at a specific point in time.

**Table 1:** Actual score, level of satisfaction (% of maximum possible score) and mean score in each subscale of the questionnaire

***Subscale***	***No. items***	***Maximum possible score***	***Actual score***	***Level of satisfaction (%)***	***Mean score x (SD)***
Healthy and safe working conditions	3	15	12.54	83.60	4.18(0.69)
Superior-subordinate communication	5	25	20.43	81.72	4.09(0.74)
Organisation of the working activity	5	25	19.88	79.52	3.98(0.80)
Teamwork	2	10	7.65	76.50	3.82(1.11)

Data were collected only from present workers and excluded those that were absent for health reasons. We did not ask our respondents about pay satisfaction since we speculated that they, being of higher social standing, would be more concerned about other factors such as communication with their superiors, peers and workload. Furthermore, the study is based on only one institution. Therefore similarly designed studies should be conducted in other universities in order to clarify whether the collected data from various universities will present a different scenario.
